# Inflammatory responses of C57BL/6NKorl mice to dextran sulfate sodium-induced colitis: comparison between three C57BL/6 N sub-strains

**DOI:** 10.1186/s42826-021-00084-2

**Published:** 2021-01-28

**Authors:** Sou Hyun Kim, Doyoung Kwon, Seung Won Son, Tae Bin Jeong, Seunghyun Lee, Jae-Hwan Kwak, Joon-Yong Cho, Dae Youn Hwang, Min-Soo Seo, Kil Soo Kim, Young-Suk Jung

**Affiliations:** 1 College of Pharmacy, Pusan National University, Busan, 46241 South Korea; 2 College of Pharmacy, Brain Busan 21 Plus Program, Kyungsung University, Busan, South Korea; 3 Exercise Biochemistry Laboratory, Korea National Sport University, Seoul, South Korea; 4 Department of Biomaterials Science, College of Natural Resources & Life Science/Life and Industry Convergence Research Institute, Pusan National University, Miryang, South Korea; 5 Laboratory Animal Center, Daegu-Gyeongbuk Medical Innovation Foundation, Daegu, South Korea; 6 College of Veterinary Medicine, Kyungpook National University, Daegu, South Korea

**Keywords:** C57BL/6NKorl, Inflammatory bowel disease, Dextran sulfate sodium, Colitis, Inflammation

## Abstract

**Background:**

Inflammatory bowel disease (IBD), including both Crohn’s disease and ulcerative colitis, are chronic human diseases that are challenging to cure and are often unable to be resolved. The inbred mouse strain C57BL/6 N has been used in investigations of IBD as an experimental animal model. The purpose of the current study was to compare the inflammatory responsiveness of C57BL/6NKorl mice, a sub-strain recently established by the National Institute of Food and Drug Safety Evaluation (NIFDS), with those of C57BL/6 N mice from two different sources using a dextran sulfate sodium (DSS)-induced colitis model.

**Results:**

Male mice (8 weeks old) were administered DSS (0, 1, 2, or 3%) in drinking water for 7 days. DSS significantly decreased body weight and colon length and increased the colon weight-to-length ratio. Moreover, severe colitis-related clinical signs including diarrhea and rectal bleeding were observed beginning on day 4 in mice administered DSS at a concentration of 3%. DSS led to edema, epithelial layer disruption, inflammatory cell infiltration, and cytokine induction (tumor necrosis factor-α, interleukin-6, and interleukin-1β) in the colon tissues. However, no significant differences in DSS-promoted abnormal symptoms or their severity were found between the three sub-strains.

**Conclusions:**

These results indicate that C57BL/6NKorl mice responded to DSS-induced colitis similar to the generally used C57BL6/N mice, thus this newly developed mouse sub-strain provides a useful animal model of IBD.

## Background

Inflammatory bowel disease (IBD), including both Crohn’s disease and ulcerative colitis (UC), is a chronic gastrointestinal disorder with rising global incidence and prevalence [1, 2]. IBD is a lifelong disease that can occur between adolescence and adulthood, and severe progress of IBD can be life-threatening [1, 2]. The common clinical outcomes of IBD include weight loss, abdominal pain, diarrhea, and rectal bleeding [1, 2]. IBD is characterized by intestinal inflammation resulting from damaged epithelium that leads to abnormal infiltrations of gut microbiota and immune cells [1, 2]. Genetic, environmental, and gut microbial factors have been suggested to influence the development of IBD, but the exact reason for its incidence and progression remains unclear [1, 2].

Animal models for investigating IBD have been developed, and several chemicals such as dextran sulfate sodium (DSS), trinitrobenzene sulfonic acid, oxazolone, and acetic acid have been administered to rodents to induce colitis [3, 4]. Among them, the DSS-promoted mouse colitis model has been commonly used in IBD research due to its simplicity, rapid onset of symptoms, controllability, and reproducibility [3]. Supplementation of DSS (2.5–10%) in drinking water for 3 to 7 days easily produces colitis in mice, and the clinical symptoms and morphological/pathophysiological changes in the intestine are similar to those in human UC [3, 4].

The C57BL/6 inbred mouse strain is widely used in biomedical research for immunology, nutrition, and human diseases such as obesity and cancers [5, 6]. This mouse strain has been frequently used in DSS-induced IBD models and reported to be more appropriate for inducing chronic colitis compared to the BALB/c mouse strain [7]. The C57BL/6 strain can be divided into several sub-strains including C57BL/6 J and C57BL/6 N which have been bred and maintained by different vendors [5, 6]. Although both strains of inbred mice originated from identical genetic backgrounds, several genotypic and phenotypic differences between the sub-strains have been observed [5, 6, 8].

Recently, a new sub-strain of C57BL/6 N mice, named C57BL/6NKorl, was established by the National Institute of Food and Drug Safety Evaluation (NIFDS) in Korea. To identify their characteristics, the physical and biological traits of these mice have been tested and compared with those of the previously used C57BL/6 N mice [9–14]. However, the inflammatory responses of this sub-strain to DSS-induced colitis have not yet been examined. Thus, the current study analyzed the clinical signs and intestinal inflammation of C57BL/6NKorl mice treated with DSS, and the usefulness of this sub-strain in IBD research was evaluated through comparison with C57BL/6 N mice from two different suppliers.

## Results

### Effect of DSS on body weights of C57BL/6 N mice

**Fig. 1:**
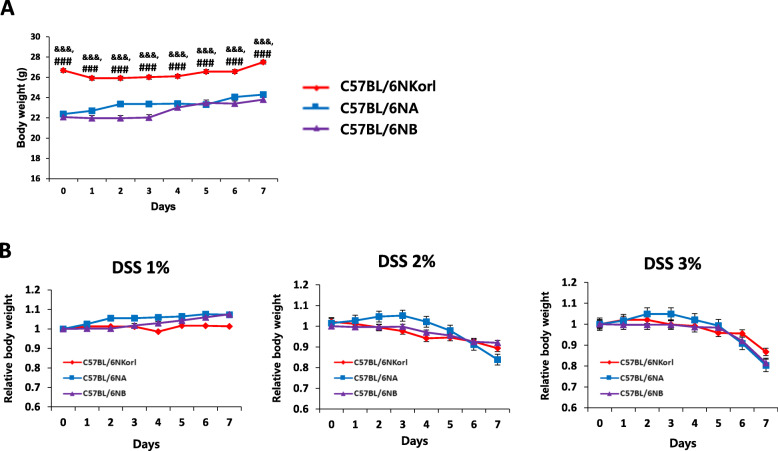
Changes in body weight three sub-strains of C57BL/6 N mice during 7 days of DSS supplementation. **a** Absolute body weight comparisons between C57BL/6NKorl and other C57BL6/N mice without DSS treatment. **b** Relative body weight changes during DSS feeding. Male C57BL/6 N mice (8-weeks-old) were given DSS (1, 2%, or 3%) in drinking water for 7 days. Differences in the relative body weight changes between the sub-strains were not found. Each value represents the mean ± SE for six mice. Student’s *t*-test, ^&&&^*P* < 0.001 vs. C57BL/6NA. ^###^P < 0.001 vs. C57BL/6NB

Prior to DSS treatment, C57BL/6NKorl mice had higher body weights compared to the other two sub-strains of C57BL/6 N mice (Fig. 1a), in agreement with previous studies [9, 10, 12, 14]. DSS caused dose- and time-dependent body weight loss (Fig. 1b) particularly in mice provided with 3% of DSS for 7 days that had a decrease of approximately 20%. There was no reduction in body weight in control mice (Fig. 1a), and no significant differences in the relative body weight change between the sub-strains overall (Fig. 1b).

### Evaluation of colitis progression by scoring the disease activity index (DAI)

**Fig. 2:**
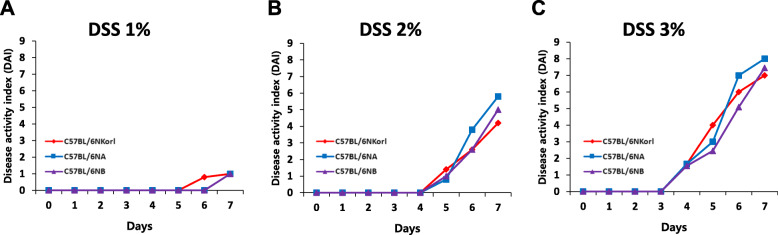
DSS-caused abnormal clinical symptoms were scored using the disease activity index (DAI). Male C57BL/6 N mice (8-weeks-old) were administered with (**a**) 1%, (**b**) 2%, or (**c**) 3% of DSS in drinking water for 7 days. DAI scores indicate the severity of abnormal signs including body weight loss, diarrhea, and rectal bleeding. Differences in the DAI scores between the sub-strains were not found

The clinical symptoms of mice with DSS-induced colitis were estimated by analyzing the DAI scoring system suggested by Cooper et al. [15]. The DAI was assessed by scoring three major physical endpoints: bodyweight loss, diarrhea, and rectal bleeding (Table 1). The scores of DAI remained unchanged in all groups until day 3, and started to increase from 3% of the DSS on day 4 (Fig. 2c). Significant body weight loss, watery and bloody stools were observed in the highest dose groups (3% DSS) (Fig. 2c), indicating severe progression of colitis. No differences between the three mice groups from different sources were found (Fig. 2).

**Table 1:** Score parameters for disease activity index (DAI)

Score	Stool consistency	Rectal bleeding	Weight loss	Maximum score
0	Normal	Normal color stool	No weight loss	10
1	Mildly soft	Brown color stool	5~10% weight loss	
2	Very soft	Reddish color stool	11~15% weight loss	
3	Watery stool	Bloody stool	16~20% weight loss	
4			>20% weight loss

### Changes in length and relative weight/length of colons of DSS-treated C57BL/6 N mice

**Fig. 3:**
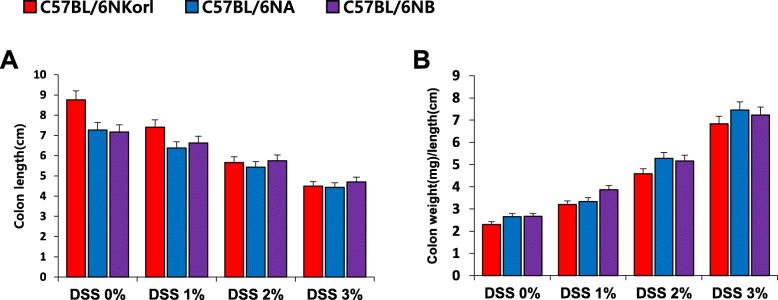
DSS-induced colonic inflammation in C57BL/6 N mice originating from three different sources. **a** Colon length. **b** Relative ratio of weight-to-length of the colon. Male C57BL/6 N mice (8 weeks old) were supplemented with DSS (0, 1, 2%, or 3%) in drinking water for 7 days. Each value represents the mean ± SE for six mice. Student’s *t*-test. Differences in the colon length and the ratio of colon weight/length between the sub-strains were not found

The mice treated with DSS (0, 1, 2, and 3%) showed a dose-dependent decreases in colon length compared to control mice (Fig. 3a). However, the ratio between the weight (mg) and length (cm) of the colon, an indirect indicator of edema and inflammation, increased in a DSS dose-dependent manner (Fig. 3b). The colonic changes in C57BL/6NKorl mice were similar to those in the other two mouse groups (Fig. 3).

### DSS-induced histopathological changes in the colon tissues of C57BL6N mice

**Fig. 4:**
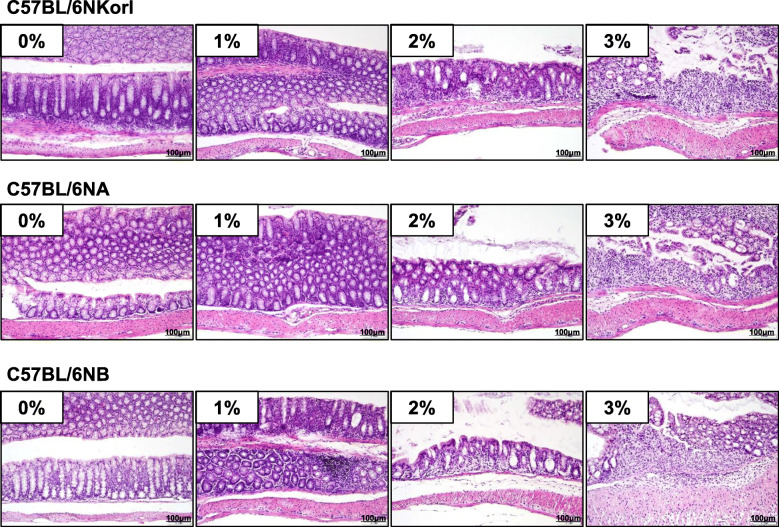
Histopathological changes in the colon tissues of DSS-treated C57BL/6 N mice. Male mice (8 weeks old) were supplemented with DSS (0, 1, 2%, or 3%) in drinking water for 7 days. Sectioned colon tissues were stained with hematoxylin and eosin (H&E) and observed at 200x magnification. Differences in the colon tissue histopathology between the sub-strains were not found

Colon tissues were stained using H&E to observe the histopathological changes in the epithelial layers. The microscopic images indicate that DSS induced edema, mucosal erosions, and disrupted epithelial layers (Fig. 4). In the 3% of DSS groups, the intestinal barrier was almost lost at 7 d (Fig. 4). Significant infiltration of inflammatory cells including lymphocytes and granulocytes was found in the submucosal and mucosal layers (Fig. 4). These results indicate that DSS promoted colonic inflammation via the loss of epithelial barrier function, but differences between the three different C57BL/6 N sub-strains were not evident (Fig. 4).

### Colonic expression of inflammatory cytokines in DSS-treated C57BL/6 N mice

**Fig. 5:**
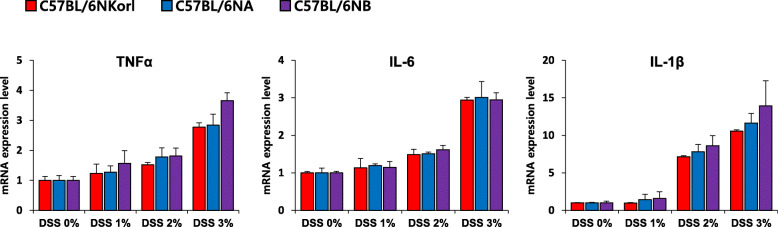
mRNA expression of inflammatory cytokines in DSS-treated C57BL/6 N mice. Male C57BL/6 N mice (8 weeks old) were supplemented with DSS (0, 1, 2%, or 3%) in drinking water for 7 days. Each value represents the mean ± SE for six mice. Differences in the cytokine expressions between the sub-strains were not found

To identify the molecular mechanism of inflammation, we examined mRNA expression of TNF-α, IL-6, and IL-1β. The three cytokines were increased depending on the concentration of DSS (Fig. 5). TNF-α and IL-6 were induced by approximately 3-fold, and IL-1β expression was elevated more than 10-fold at day 7 in all the three sub-strains of mice fed with 3% DSS (Fig. 5).

## Discussion

In the present study, DSS (40 kDa) feeding for 7 days showed severe outcomes of colitis and intestinal inflammation via marked damages in the mucosal and epithelial layers in all the three sub-strains of C57BL/6 N mice. DSS is a sulfate polysaccharide which has a wide range of molecular weights from 5 to 1400 kDa, and 36 to 50 kDa of DSS is usually employed in mice for inducing colitis [3]. The exact mechanism of DSS-promoted colitis has been not well understood, though the negatively charged sulfate groups are thought to damage the intestinal mucosa and epithelial cells, resulting in the increase of gut permeability [3, 4]. Gut microorganisms and their derivatives can penetrate through the loosened epithelial tight junctions, activating intestinal macrophages which then secrete inflammatory cytokines including TNF-α, IL-1β, and IL-6 [1, 2]. The cytokines act as chemokines that recruit other immune cells such as dendritic cells, T cells, B cells, and neutrophils which aggravate colonic inflammation [1, 2]. The gastrointestinal disorders of IBD also affect the absorption of nutrients which can cause malnutrition and anemia [1, 2].

A new inbred sub-strain can be developed by consecutive sib-mating for more than 20 generations using the offspring from a single breeding pair. C57BL/6 N mice were originally established in the National Institutes of Health (NIH, Bethesda, MD, USA) using C57BL/6 J mice which were transferred from The Jackson Laboratory (Bar Harbor, ME, USA) in the 1950s. C57BL/6NKorl mice were recently developed in Korea under a project by the Ministry of Food and Drug Safety for the localization of experimental animal resources [5]. After 33 generations of inbreeding from 2005, C57BL/6NKorl was established and certified as a unique sub-strain by the Institute for Laboratory Animal Research (ILAR) in 2015 [5, 9].

Genetic differences between inbred mouse sub-strains can be produced by residual heterozygosity and spontaneous mutations [16]. The characteristics of C57BL/6NKorl have been identified by comparing these mice to the generally used C57BL/6 N sub-strains from different sources. The C57BL/6NKorl mice have shown similar humoral immunity, fertilization and embryo development rates, tumorigenesis, and responses to cisplatin and restraint stress compared to other C57BL/6 N mice [9–14]. However, relatively higher body weight and adipose tissue mass, and lower oxygen consumption were found to be distinct features of the C57BL/6NKorl sub-strain [9, 10, 12, 14]. Moreover, cell-mediated immune response of C57BL/6NKorl mice determined by concanavalin A-induced splenic T cell proliferation was stronger than those of other C57BL/6 N sub-strains [11]. In the present study, despite the relatively higher body weights of C57BL/6NKorl mice, there were no differences in the symptoms and colonic responses in DSS-induced colitis between the three C57BL/6 N sub-strains, suggesting that the newly generated C57BL/6NKorl mice can be used as an animal model of IBD.

## Conclusions

In the present study, we found that IBD was easily induced in C57BL/6NKorl mice by using the general protocol of the DSS colitis model, and this sub-strain showed similar inflammatory responses and pathophysiological features in comparison with other C57BL/6 N sub-strains. Based on the positive responsiveness to DSS, this new sub-strain provides a valuable animal resource for investigating IBD. However, age- and sex-dependent alterations and differences in DSS colitis and the biological responses of other IBD models remain unknown. Therefore, further studies are needed to further clarify the exact characteristics of C57BL/6NKorl mice.

## Methods

### Animals and treatments

Male C57BL/6 N mice (8-week-old) were obtained from three different suppliers. The C57BL/6NKorl mice (*n* = 24) were provided by the Department of Laboratory and Animal Resources at the National Institute of Food and Drug Safety Evaluation (NIFDS, Cheongju, Korea). The other two groups of C57BL/6 N mice were purchased from different vendors located in the United States (referred to as C57BL/6NA; n = 24) and Japan (referred to as C57BL/6NB; n = 24). Experimental protocols were approved by the Institutional Animal Care and Use Committee of Pusan National University (Approval Number: PNU-2019-2230). Mice were acclimated to the University’s animal facility for 1 week prior to the experiment at standard room temperature (22 ± 2 °C) and humidity (55 ± 5%) with a 12-h light/dark cycle.

### Induction of DSS-induced ulcerative colitis

Mice (n = 24 from each source) were randomly divided into 4 groups. Colitis was induced by providing the mice with 0% (*n* = 6), 1% (n = 6), 2% (n = 6), or 3% (n = 6) of DSS (molecular weight, 40 kDa; ICN Biomedicals Inc., Cleveland, OH, USA) in drinking water for 7 days. Body weights were recorded daily. The disease activity index (DAI) was determined by scoring body weight loss, stool consistency, and rectal bleeding according to the scoring system suggested by Cooper et al. [15]. All parameters detailed in Table 1 were examined and scored from day 0 to day 7 during the DSS treatment. Collected colon tissue was washed in phosphate-buffered saline (PBS), and the length and weight were measured to calculate the weight-to-length ratio.

### Histopathological analysis

Colon tissues were fixed with 4% neutral buffered formalin. Tissues were embedded in paraffin, and a 5-μm section was stained with hematoxylin and eosin (H&E) to discriminate between the nuclei and cytoplasm.

### RNA purification and quantitative reverse transcription polymerase chain reaction (RT-PCR)

Total RNA was isolated from the colon lysate using the Direct-zol RNA kit (Zymo Research, Orange, CA, USA). The cDNA was synthesized using the iScript cDNA Synthesis System (Bio-Rad, Hercules, CA, USA). Quantitative RT-PCR was performed using the SensiFAST SYBR qPCR mix (Bioline, London, UK) according to the manufacturer’s protocol. The primer sequences of tumor necrosis factor-α (TNF-α), interleukin-6 (IL-6), interleukin-1β (IL-1β), and 18S ribosomal RNA (18 s) used in this study are reported in Table 2. The values of gene expression were normalized to those of 18 s.

**Table 2:** List of primers used for real time RT-PCR

Symbol	Full name	Primer sequence (5’-3’)	
		Forward	Reverse
*Tnf-α*	Tumor necrosis factor-α	GGCCTCTCTACCTTGTTGCC	CAGCCTGGTCACCAAATCAG
*Il-6*	Interleukin-6	TTGCCTTCTTGGGACTGATG	CCACGATTTCCCAGAGAACA
*Il-1β*	Interleukin-1β	TTCACCATGGAATCCGTGTC	GTCTTGGCCGAGGACTAAGG
*18S*	18S ribosomal RNA	CAGCCACCCGAGATTGAGCA	TAGTAGCGACGGGCGGTGTG

### Statistical analysis

All results are expressed as mean ± standard error (SE). Tests of significance were performed using a Student’s *t*-test, with a *P*-value < 0.05 set for significance.

## Data Availability

The datasets used and/or analyzed in this study are available from the corresponding author on reasonable request.

## Abbreviations

DAI: Disease activity index
DSS: Dextran sulfate sodium
IBD: Inflammatory bowel disease
IL-1β: Interleukin-1β
IL-6: Interleukin-6
NIFDS: National Institute of Food and Drug Safety Evaluation
TNF-α: Tumor necrosis factor-α
UC: Ulcerative colitis
